# Supplementary material for: Between-trial heterogeneity in meta-analyses may be partially explained by reported design characteristics
Source: J Clin Epidemiol. 2018 Mar;95:45–54. doi: 10.1016/j.jclinepi.2017.11.025 (PMC5828111; doi:10.1016/j.jclinepi.2017.11.025)
Supplement: Table S1 [file mmc1.docx]

**Table S1** Posterior mean residual deviance *D_res_*, effective number of parameters *p_D_* and deviance information criterion (DIC) for the hierarchical models fitted to the ROBES data.

| **Model** | **Design characteristic/s** | **Interaction/s between design characteristics** | **Covariates in model for *τ^2^*** | ***D_res_*** | ***p_D_*** | **DIC** |
| --- | --- | --- | --- | --- | --- | --- |
| A1 | Sequence generation | N/A | - | 2982 | 1909 | 4891 |
|  | Sequence generation | N/A | Outcome type | 3000 | 1889 | 4889 |
| A2 | Allocation concealment | N/A | - | 2972 | 1914 | 4886 |
|  | Allocation concealment | N/A | Outcome type | 3003 | 1899 | 4902 |
| A3 | Blinding | N/A | - | 2968 | 1915 | 4883 |
|  | Blinding | N/A | Outcome type | 3003 | 1891 | 4894 |
| B1 | Sequence generation and allocation concealment | Yes | - | 3001 | 1900 | 4901 |
|  | Sequence generation and allocation concealment | No | - | 2988 | 1906 | 4894 |
| B2 | Sequence generation and blinding | Yes | - | 2978 | 1908 | 4886 |
|  | Sequence generation and blinding | No | - | 2988 | 1904 | 4892 |
| B3 | Allocation concealment and blinding | Yes | - | 2996 | 1902 | 4898 |
|  | Allocation concealment and blinding | No | - | 2981 | 1908 | 4889 |
| B4 | Sequence generation, allocation concealment and blinding | All possible | - | 2985 | 1905 | 4890 |
|  | Sequence generation, allocation concealment and blinding | Interaction between sequence generation and blinding alone | - | 2998 | 1899 | 4897 |
|  | Sequence generation, allocation concealment and blinding | No | - | 2978 | 1913 | 4891 |
|  | Sequence generation, allocation concealment and blinding | No | Outcome type | 2991 | 1895 | 4886 |
