# Supplementary material for: Between-trial heterogeneity in meta-analyses may be partially explained by reported design characteristics
Source: J Clin Epidemiol. 2018 Mar;95:45–54. doi: 10.1016/j.jclinepi.2017.11.025 (PMC5828111; doi:10.1016/j.jclinepi.2017.11.025)
Supplement: Supplementary Materials [file mmc2.docx]

**Supplementary materials**

**S1 Estimating total heterogeneity variance from the label-invariant model**

We used label-invariant hierarchical models to analyse trial data from 117 meta-analyses in *ROBES* simultaneously. The models have been proposed in an earlier paper [9], but we describe the models briefly here to show how to derive the formulae for heterogeneity variance $\tau_{total,m}^{2}$ among all trials in a meta-analysis *m*.

**S1.1 Univariable model for the influence of** accounting for **a single trial design characteristic**

In a given meta-analysis *m*, trials are categorised as low risk of bias (L-trials) or high/unclear risk of bias (H-trials) for a specific design characteristic.

The L-trials provide an estimate of the underlying intervention effect $\theta_{im}^{L}$, assumed to have a normal random-effects distribution with mean $d_{m}$ and variance $\tau_{m}^{2}$, specific to meta-analysis *m*. The H-trials are assumed to estimate an underlying intervention effect $\theta_{im}^{H}$, assumed to be normally distributed with mean$d_{m}+b_{m}$and variance $\lambda\tau_{m}^{2}$:

$$\begin{matrix} \theta_{im}^{L}\sim N(d_{m},\tau_{m}^{2}) \\ \theta_{im}^{H}\sim N(d_{m}+b_{m},\lambda\tau_{m}^{2}). \end{matrix}$$

The average bias *b_m_* in intervention effect in meta-analysis *m* is assumed to be exchangeable across meta-analyses, with overall mean *b_0_* and between-meta-analysis variance in mean bias $\text{φ}^{2}$:

We set an indicator *X_im_* to be 1 for H trials and 0 for L trials such that

$$X_{im}=\left\{\begin{matrix} 1 & & \pi_{m} \\ & \text{with probability} & \\ 0 & & 1-\pi_{m}. \end{matrix}\right.$$

Each trial is assumed to provide an underlying estimate of intervention effect:

$\theta_{im}=(1-X_{im})\theta_{im}^{L}+X_{im}\theta_{im}^{H}$.

The first term of the sum will return $\theta_{im}^{L}$ if trial *i* is at low risk of bias. The second term will return $\theta_{im}^{H}$ if the trial *i* is at high/unclear risk of bias.

The total heterogeneity variance among trials in meta-analysis *m* is given by:

$$\begin{matrix} \tau_{total,m}^{2}=\mathrm{var}(\theta_{im}) \\ =\mathrm{var}((1-X_{im})\theta_{im}^{L}+X_{im}\theta_{im}^{H}) \\ =\mathrm{var}((1-X_{im})\theta_{im}^{L})+\mathrm{var}(X_{im}\theta_{im}^{H})+2\mathrm{cov}((1-X_{im})\theta_{im}^{L},X_{im}\theta_{im}^{H}) \end{matrix}$$

$$\begin{matrix} =E(1-X_{im})^{2}\mathrm{var}(\theta_{im}^{L})+E(\theta_{im}^{L})^{2}\mathrm{var}(1-X_{im})+\mathrm{var}(1-X_{im})\mathrm{var}(\theta_{im}^{L}) \\ +E(X_{im})^{2}\mathrm{var}(\theta_{im}^{H})+E(\theta_{im}^{H})^{2}\mathrm{var}(X_{im})+\mathrm{var}(X_{im})\mathrm{var}(\theta_{im}^{H}) \\ +2[E((1-X_{im})\theta_{im}^{L}X_{im}\theta_{im}^{H})-E((1-X_{im})\theta_{im}^{L})E(X_{im}\theta_{im}^{H})] \end{matrix}$$

$$\begin{matrix} =E(1-X_{im})^{2}\mathrm{var}(\theta_{im}^{L})+E(\theta_{im}^{L})^{2}\mathrm{var}(1-X_{im})+\mathrm{var}(1-X_{im})\mathrm{var}(\theta_{im}^{L}) \\ +E(X_{im})^{2}\mathrm{var}(\theta_{im}^{H})+E(\theta_{im}^{H})^{2}\mathrm{var}(X_{im})+\mathrm{var}(X_{im})\mathrm{var}(\theta_{im}^{H}) \\ +2[E((1-X_{im})X_{im})E(\theta_{im}^{L}\theta_{im}^{H})-E((1-X_{im})\theta_{im}^{L})E(X_{im}\theta_{im}^{H})] \end{matrix}$$

$$\begin{matrix} =(1-\pi_{m})^{2}\tau_{m}^{2}+d_{m}^{2}(1-\pi_{m})\pi_{m}+(1-\pi_{m})\pi_{m}\tau_{m}^{2} \\ +\pi_{m}^{2}\lambda\tau_{m}^{2}+(d_{m}+b_{m})^{2}\pi_{m}(1-\pi_{m})+\pi_{m}(1-\pi_{m})\lambda\tau_{m}^{2} \\ -2(1-\pi_{m})d_{m}\pi_{m}(d_{m}+b_{m}) \end{matrix}$$

$$\begin{matrix} =(1-\pi_{m})\tau_{m}^{2}+d_{m}^{2}(1-\pi_{m})\pi_{m} \\ +\pi_{m}\lambda\tau_{m}^{2}+(d_{m}+b_{m})^{2}\pi_{m}(1-\pi_{m}) \\ -2(1-\pi_{m})d_{m}\pi_{m}(d_{m}+b_{m}) \\ \\ =(1-\pi_{m})\tau_{m}^{2}+\pi_{m}\lambda\tau_{m}^{2}+\pi_{m}(1-\pi_{m})b_{m}^{2} \end{matrix}$$

**S1.2 Multivariable model for the influence of** accounting for **multiple trial design characteristics**

Suppose trials in a meta-analysis *m* are categorised as low risk of bias (L-trials) or high/unclear risk of bias (H-trials) for each of 2 reported design characteristics. We set the indicator *X_ijm_* to be 1 for trials at high/unclear risk of bias for the *j*-th reported characteristic (*j=*1,2), and 0 for trials at low risk of bias for that characteristic such that

$$X_{ijm}=\left\{\begin{matrix} 1 & & \pi_{jm} \\ & \text{with probability} & \\ 0 & & 1-\pi_{jm} \end{matrix}\right.$$

Each trial is assumed to provide an estimate of underlying intervention effect:

$$\theta_{im}=(1-X_{1im})(1-X_{2im})\theta_{im}^{L}+X_{1im}(1-X_{2im})\theta_{1im}^{H}+X_{2im}(1-X_{1im})\theta_{2im}^{H}+X_{1im}X_{2im}\theta_{3im}^{H}$$

where

$$\begin{matrix} \theta_{im}^{L}\sim N(d_{m},\tau_{m}^{2}) \\ \theta_{1im}^{H}\sim N(d_{m}+b_{1m},\lambda_{1}\tau_{m}^{2}) \\ \theta_{2im}^{H}\sim N(d_{m}+b_{2m},\lambda_{2}\tau_{m}^{2}) \\ \theta_{3im}^{H}\sim N(d_{m}+b_{1m}+b_{2m}+b_{3m},\lambda_{1}\lambda_{2}\lambda_{3}\tau_{m}^{2}). \end{matrix}$$

Trials at low risk of bias for both characteristics 1 and 2 provide an estimate of intervention effect $\theta_{im}^{L}$, as in Section S1.1. The intervention effect $\theta_{1im}^{H}$ in a trial *i* at high/unclear risk of bias for characteristic 1 but low risk of bias for characteristic 2 has a normal distribution with mean $\text{d}_{\text{m}}\text{+b}_{\text{1m}}$ and variance $\text{τ}_{\text{m}}^{2}\text{λ}_{1}.$ The intervention effect $\theta_{2im}^{H}$ in a trial *i* at high/unclear risk of bias for characteristic 2 but low risk of bias for characteristic 1 has a normal distribution with mean $\text{d}_{\text{m}}\text{+b}_{\text{2m}}$ and variance $\text{τ}_{\text{m}}^{2}\text{λ}_{2}.$ The intervention effect $\theta_{3im}^{H}$ in a trial *i* at high/unclear risk of bias for both characteristics 1 and 2 has a normal distribution with mean $\text{d}_{\text{m}}\text{+b}_{\text{1m}}\text{+b}_{\text{2m}}+b_{3m}$ and variance $\text{τ}_{\text{m}}^{2}\text{λ}_{1}\text{λ}_{2}\text{λ}_{3}.$

An estimate of total heterogeneity variance among trials in meta-analysis *m* is given by:

$$\begin{matrix} \tau_{total,m}^{2}=\mathrm{var}(\theta_{im}) \\ =\mathrm{var}((1-X_{1im})(1-X_{2im})\theta_{im}^{L}+X_{1im}(1-X_{2im})\theta_{1im}^{H}+X_{2im}(1-X_{1im})\theta_{2im}^{H}+X_{1im}X_{2im}\theta_{3im}^{H}) \\ =\mathrm{var}((1-X_{1im})(1-X_{2im})\theta_{im}^{L})+\mathrm{var}(X_{1im}(1-X_{2im})\theta_{1im}^{H})+\mathrm{var}(X_{2im}(1-X_{1im})\theta_{2im}^{H})+\mathrm{var}(X_{1im}X_{2im}\theta_{3im}^{H}) \\ +2\mathrm{cov}((1-X_{1im})(1-X_{2im})\theta_{im}^{L},X_{1im}(1-X_{2im})\theta_{1im}^{H})+2\mathrm{cov}((1-X_{1im})(1-X_{2im})\theta_{im}^{L},X_{2im}(1-X_{1im})\theta_{2im}^{H}) \\ +2\mathrm{cov}((1-X_{1im})(1-X_{2im})\theta_{im}^{L},X_{1im}X_{2im}\theta_{3im}^{H})+2\mathrm{cov}(X_{1im}(1-X_{2im})\theta_{1im}^{H},X_{2im}(1-X_{1im})\theta_{2im}^{H}) \\ +2\mathrm{cov}(X_{1im}(1-X_{2im})\theta_{1im}^{H},X_{1im}X_{2im}\theta_{3im}^{H})+2\mathrm{cov}(X_{2im}(1-X_{1im})\theta_{2im}^{H},X_{1im}X_{2im}\theta_{3im}^{H}) \\ \\ =(1-\pi_{1m})(1-\pi_{2m})\tau_{m}^{2}+((1-\pi_{1m})(1-\pi_{2m})-(1-\pi_{1m})^{2}(1-\pi_{2m})^{2})d_{m}^{2} \\ +\pi_{1m}(1-\pi_{2m})\lambda_{1}\tau_{m}^{2}+(\pi_{1m}(1-\pi_{2m})-\pi_{1m}^{2}(1-\pi_{2m})^{2})(d_{m}+b_{1m})^{2} \\ +\pi_{2m}(1-\pi_{1m})\lambda_{2}\tau_{m}^{2}+(\pi_{2m}(1-\pi_{1m})-\pi_{2m}^{2}(1-\pi_{1m})^{2})(d_{m}+b_{2m})^{2} \\ +\pi_{1m}\pi_{2m}\lambda_{1}\lambda_{2}\lambda_{3}\tau_{m}^{2}+(\pi_{1m}\pi_{2m}-\pi_{1m}^{2}\pi_{2m}^{2})(d_{m}+b_{1m}+b_{2m}+b_{3m})^{2} \\ -2\pi_{1m}(1-\pi_{1m})(1-\pi_{2m})^{2}d_{m}(d_{m}+b_{1m}) \\ -2\pi_{2m}(1-\pi_{2m})(1-\pi_{1m})^{2}d_{m}(d_{m}+b_{2m}) \\ -2\pi_{1m}\pi_{2m}(1-\pi_{1m})(1-\pi_{2m})d_{m}(d_{m}+b_{1m}+b_{2m}+b_{3m}) \\ -2\pi_{1m}(1-\pi_{2m})\pi_{2m}(1-\pi_{1m})(d_{m}+b_{1m})(d_{m}+b_{2m}) \\ -2\pi_{1m}^{2}(1-\pi_{2m})\pi_{2m}(d_{m}+b_{1m})(d_{m}+b_{1m}+b_{2m}+b_{3m}) \\ -2\pi_{2m}^{2}(1-\pi_{1m})\pi_{1m}(d_{m}+b_{2m})(d_{m}+b_{1m}+b_{2m}+b_{3m}). \end{matrix}$$

In a similar way, we derive estimates of total heterogeneity in a meta-analysis from the multivariable label-invariant models for the influence of accounting for three design characteristics.

**S2 Model comparison**

Bayesian hierarchical models were fitted to trial data from all 117 meta-analyses. The various models fitted to the data differed according to the indicators of design characteristics and interactions included as covariates in the model, and according to the inclusion of indicators of outcome type in the regression model for heterogeneity variance $\tau_{m}^{2}$ among trials at low risk of bias. Results to compare model fit are given in Table S1.
